# Advances in Medical Devices for Augmented Acupuncture Therapy

**DOI:** 10.1002/advs.202505318

**Published:** 2025-07-18

**Authors:** Ruisi Cai, Chenyi Yu, Xinyi Luo, Ningyu Xu, Jicheng Yu, Xiao Ye, Yuqi Zhang, Zhen Gu

**Affiliations:** ^1^ State Key Laboratory of Advanced Drug Delivery and Release Systems Zhejiang Provincial Key Laboratory for Advanced Drug Delivery Systems School of Pharmacy Zhejiang University Hangzhou 310058 China; ^2^ Pharmaceutical Sciences Shandong University Jinan Shandong 250012 China; ^3^ Jinhua Institute of Zhejiang University Jinhua 321299 China; ^4^ Department of General Surgery, Sir Run Run Shaw Hospital School of Medicine Zhejiang University Hangzhou 310016 China; ^5^ Center for General Practice Medicine, Department of Endocrinology Zhejiang Provincial People's Hospital, Affiliated People's Hospital Hangzhou medical college Hangzhou 310014 China; ^6^ Key Laboratory for Diagnosis and Treatment of Endocrine Gland Diseases of Zhejiang Province Hangzhou 310014 China; ^7^ Department of Burns and Wound Care Center Second Affiliated Hospital School of Medicine Zhejiang University Hangzhou 310009 China; ^8^ MOE Key Laboratory of Macromolecular Synthesis and Functionalization Department of Polymer Science and Engineering Zhejiang University Hangzhou 310027 China; ^9^ Institute of Fundamental and Transdisciplinary Research Zhejiang University Hangzhou 310058 China; ^10^ Engineering Research Center of Innovative Anticancer Drug Ministry of Education Hangzhou 310058 China

**Keywords:** acupuncture, drug delivery, engineered acupuncture needle, medical device, physical stimulation

## Abstract

Acupuncture, as a traditional medicine applied worldwide, is considered an effective treatment for numerous chronic diseases. Many researches have explored the biological mechanisms of acupuncture, including the skin‐neuro‐immune axis and inflammation‐immune modulation. However, traditional acupuncture faces several limitations, such as its complex operation, the wide variety of acupoint combinations, long treatment durations, and susceptibility to individual differences between the practitioner and the patient. These challenges highlight the urgent need for intelligent medical devices that enable more precise and efficient modern acupuncture treatment. In response to this, this manuscript introduces engineered acupuncture needles (EANs) using techniques such as macroscopic mechanical etching, microscopic surface modification, and biomedical nanotechnology. These modified needles enhance acupuncture treatment by incorporating drug delivery, electrical stimulation, and thermal stimulation technologies. Here we provide an overview of recent advancements in EANs in terms of materials, fabrication methods, engineering technologies, acupoints, as well as disease models. The relevant bottlenecks and future prospects of their development are also discussed.

## Graphical abstract

Traditional acupuncture therapy faces several challenges in clinical applications, such as complex operation procedures and prolonged therapeutic periods. By incorporating physical stimulations and drug delivery technologies, engineered acupuncture needles (EANs) can enhance therapeutic efficacy, improve patient compliance, as well as mitigate associated side effects. This perspective highlights an overview of recent advancements on EANs, focusing on developed materials, fabrication methods, engineering technologies, targeted acupoints, and disease models. The relevant bottlenecks and future prospects of their development are also discussed.

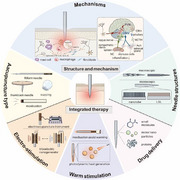

## 1 Introduction

**Figure 1:**
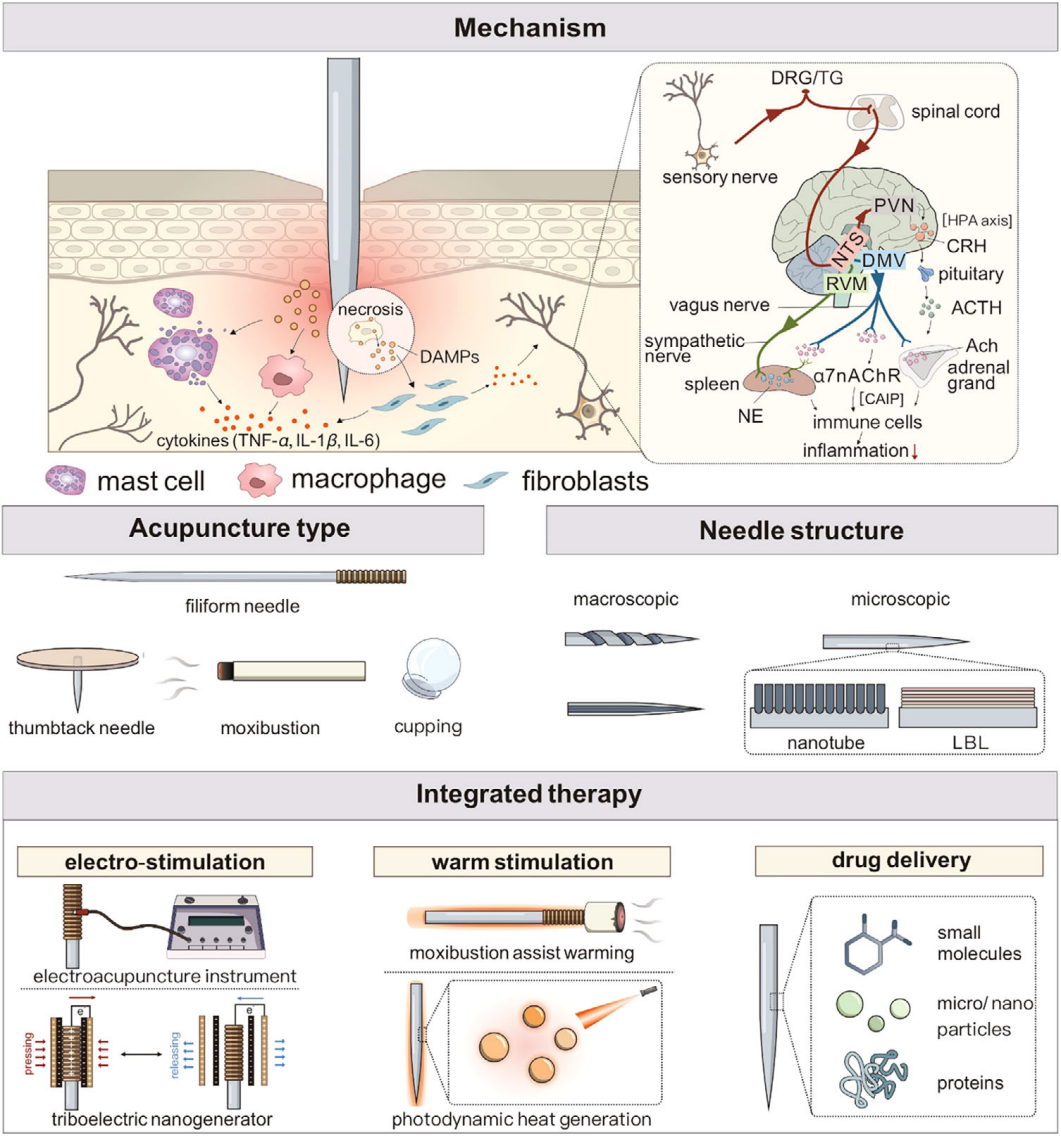
Schematic diagrams of acupuncture needles, including mechanisms, types, structures and integrated applications of therapy. DAMPs: Damage associated molecular patterns; DRG, dorsal root ganglia; TG, trigeminal ganglia; *α*7nAChR, *α*7 nicotinic acetylcholine receptors; CAIP, cholinergic anti‐inflammatory pathway; HPA axis, hypothalamic‐pituitary‐adrenal axis; NTS, nucleus tractus solitarius; RVM, rostral ventrolateral medulla; DMV, dorsal motor nucleus of vagus; PVN, paraventricular nucleus; CRH, corticotropin‐releasing hormone; ATCH, adrenocorticotropic hormone; ACh, acetylcholine; NE, noradrenaline; LBL: layer‐by‐layer assembly.

Acupuncture, as an integral component of traditional Chinese medicine, has developed over thousands of years and is widely practiced in various Asian countries and across the globe. It has demonstrated with documented therapeutic effects in pain management, neurological disorders, and systemic inflammation regulation.^[^ ^1^, ^2^, ^3^, ^4^ ^]^ Acupuncture encompasses two primary modalities: “needling” and “moxibustion”. “Needling” involves the application of conventional filiform needles, triangle‐edged needles and thumbtack needles, whereas “moxibustion” refers to the therapeutic method of applying thermal stimulation to acupoints via combustion of moxa floss‐based materials, such as moxa sticks. Additionally, cupping therapy is frequently employed as an ancillary therapeutic modality to acupuncture in clinical practice. Contemporary research indicates that, histologically, acupoints are not only located in areas with concentrated neural innervation but are also enriched in regions with high densities of mast cells, lymphatic vessels, and arteriovenous plexuses, with a depth distribution ranging from 0.5 to 5 cm.^[^ ^5^ ^]^ Acupuncture exerts anti‐inflammatory effects through mechanical stimulation, which activates the nuclear factor kappa‐B, mitogen‐activated protein kinase, and extracellular regulated protein kinases pathways in local immune cells. This process promotes the polarization of macrophages from the M1 to the M2 phenotype and regulates inflammatory cytokines, including interleukin‐6 (IL‐6), interleukin‐1*β* (IL‐1*β*) and tumor necrosis factor‐*α* (TNF‐*α*).^[^ ^6^, ^7^, ^8^, ^9^, ^10^ ^]^ Acupoint stimulation has also been shown to activate various neuroimmune pathways, including the vagus‐adrenal medulla‐dopamine and sympathetic pathways.^[^ ^11^, ^12^ ^]^ Specifically, acupuncture can stimulate mechanoreceptors, stretch receptors, and free nerve endings at the acupoint, with the resulting afferent signals transmitted to corresponding nuclei in the brainstem and hypothalamus.^[^ ^5^ ^]^ Following integration by the central nervous system, these signals induce systemic anti‐inflammatory effects through various pathways, such as the cholinergic anti‐inflammatory pathway, the vagus–adrenal medullary–dopamine axis, and sympathetic neural circuits (**Figure** **1**). For example, stimulation of acupoints such as ST36 (“Zusanli”) selectively activates the insular cortex and the hypothalamic‐pituitary‐adrenal (HPA) axis, thereby triggering systemic anti‐inflammatory and analgesic responses through neuroendocrine regulation.^[^ ^12^, ^13^, ^14^ ^]^ Based on the aforementioned mechanisms and the therapeutic efficacy observed in clinical applications, the World Health Organization has incorporated acupuncture into its Traditional Medicine Strategy (2014‐2023).

However, acupuncture still confronts multiple challenges in clinical practice, including heterogeneity in therapeutic efficacy, limitations in multidimensional modulation, and absence of standardized quantitative evaluation frameworks.^[^ ^15^, ^16^ ^]^ In detail, the therapeutic effect was highly operator‐dependent, that the techniques of traditional acupuncture and twisting vary from person to person.^[^ ^17^, ^18^ ^]^ The singular stimulation provided by traditional acupuncture needles is insufficient to meet the multi‐target regulatory demands of complex diseases, such as chronic neuropathic pain.^[^ ^19^, ^20^ ^]^ Moreover, the associated therapeutic effects could be attenuated and usually require a prolonged treatment period due to the compromised mechanical stimuli within the tissue and uncontrollable heats conducted through needles.^[^ ^21^, ^22^, ^23^ ^]^ Recently, diverse biomedical devices have been designed to enhance acupuncture therapy by integrating techniques of metal processing, biomedical nanotechnology, and intelligent electronics with acupuncture needles. These advanced engineered acupuncture needles (EANs) can be customized to achieve controllable drug delivery, programable electrostimulation, and precise heat stimulation, demonstrating high therapeutic efficiency in treating various diseases.^[^ ^16^, ^24^ ^]^

Herein, we introduce the latest advancements in EAN‐enhanced acupuncture therapy with a particular focus on the application involved electro‐conduction needles, heat‐conduction needles, and drug‐delivering needles (Figure 1). With the assistance of engineering techniques, the EANs can enhance the stimulation of the neuro‐immune axis by augmented physical stimulations, as well as targeted delivered drugs to treat diverse diseases. The materials and construction of EANs, manuduction methods, integration of physical stimulations, traditional acupoints and according disease models are summarized. Finally, the key challenges and future prospects of the development of EANs were also discussed.

## 2 Physically Strengthened Acupuncture Therapy

With the integration of traditional practices and modern technologies, thermotherapy‐ and electrotherapy‐assisted acupuncture was exploited as a powerful tool in contemporary medicine. Based on acupoint stimulation, the conduction of heat or electricity through EANs not only promotes blood circulation and metabolism but also modulates distal physiological responses by activating downstream pathways, thereby enhancing the therapeutic efficacy of traditional acupuncture.^[^ ^25^ ^]^

### 2.1 Warm Acupuncture

Warm needle acupuncture (WA), also known as warm needling or thermal acupuncture, generates heat by burning moxa attached to the needle handle, allowing thermal energy to be conducted along the needle shaft to the skin. After the WA needle is inserted to the targeted acupoints, the heat generated by the burning moxa stimulates the cutaneous thermoreceptors and penetrated through the muscles to the deep issues. The combination of mechanical stimulation from the needle and heat stimulation from moxibustion has been shown to generate physiological effects, such as elevating metabolism, enlarging blood vessels, increasing peripheral blood flow, and reducing the excitability of peripheral nerves.^[^ ^25^, ^26^ ^]^ The appearance of such physiological effects and therapeutic effects has tight connections with the temperature. The mechanism relates to temperature responsive the nerve fibers so‐called C‐fibers which transduce the thermal sensation. Alternatively, Tan et al. found that WA inhibited oxidative stress and inflammatory responses in the cartilage tissue of model rats, with the reduced expression of malondialdehyde and NADPH‐oxidase while increased expression of superoxide dismutase 2. The changed expression level of inflammation related factors indicates that the mechanism may associated with the modulation of pro‐inflammatory factors during oxidative stress.^[^ ^27^ ^]^ Although the heat transmitted by warm acupuncture has been proven to induce various beneficial physiological effects, maintaining a safe temperature and controlled heat distribution is crucial. Hyo‐Rim Jo et al. set the therapeutic temperature window of WA at 40 °C to 45 °C in their study and comparing the heat distributions between WA and heated‐needle acupuncture (HA, which directly heated by the lighter on the needle surface before applying to the skin).^[^ ^28^ ^]^ Within this temperature window, WA can produce beneficial physiological effects, such as increasing blood flow velocity and enhancing vascular permeability, while avoiding the sensation of thermal pain. This is achieved by maintaining the temperature below the threshold for nociceptor activation (A fibers), which is ≈44.4 ± 2.1 °C.^[^ ^29^, ^30^ ^]^

This technique overcomes the limitations of traditional moxibustion in delivering heat to deep pathological sites, and has been widely applied in clinical practice for pain release, osteoarthritis (OA), lumbar disc herniation, and type 2 diabetic peripheral neuropathy.^[^ ^31^ ^]^ Clinical studies have demonstrated that WA exhibits superior efficacy in pain relief and inflammation control compared to traditional acupuncture, potentially assisting conventional pharmacological treatment.^[^ ^32^ ^]^ A meta‐analysis conducted by Ji et al. evaluated the efficacy and safety of WA alone or in combination with pharmacological treatment for patients with OA. The results demonstrated that, compared to drug therapy, WA had a beneficial effect on overall efficacy (risk ratio: 1.22, 95% confidence interval: 1.17 to 1.27; 24 studies, 2278 participants). Notably, WA exhibited enhanced effects on overall efficacy when compared to celecoxib, diclofenac, and ibuprofen (risk ratio: 1.20–1.27, 95% confidence interval: 1.08 to 1.42). In terms of pain relief, WA showed a greater effect than diclofenac and ibuprofen (standardized mean difference: 3.52, 95% confidence interval: 5.90 to 1.12; 8 studies, 694 participants).^[^ ^33^ ^]^

### 2.2 Electroacupuncture

**Figure 2:**
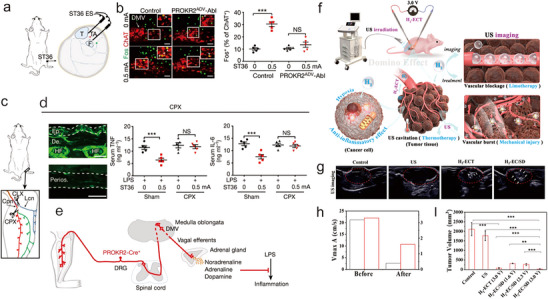
Mechanisms and applications of electromediated EANs. a) Schematic of electrostimulation at the ST36. ES: electrostimulation. b) Images (left) and quantification (right) of Fos expression (green) induced by electrostimulation (0.5 mA) at the ST36 acupoint in ChAT+ (red) DMV neurons in control but not in PROKR2ADV‐Abl mice. c) Schematic of common peroneal nerve (Cpn) neurectomy (CPX) and lateral cutaneous nerve (Lcn) neurectomy (CLX). d) CPX mice showed preservation of TUBB3+ fibres (green) in skin at the ST36 region (top), but loss in the tibial periosteum (bottom), scale bar: 100 µm; right: electrostimulation (0.5 mA) at ST36 reduced LPS‐induced production of TNF and IL‐6 in mice that underwent sham surgery compared with CPX. e) Schematic of the vagal–adrenal axis driven by PROKR2Cre neurons innervating deep limb fascial tissues. Adapted with permission.^[^ ^13^ ^]^ Copyright 2021, Springer Nature. f) Diagram exhibiting imaging guided endogenic H_2_‐augmented electrochemo‐sonodynamic domino co‐therapy. g) The generated H_2_ promoted acupuncture localization (the white arrowhead indicated H_2_ bubbles). h) The blood flow speed changed before and after the H_2_‐EC/SD (3.0 V). i) Tumor volume under different treatment. Adapted with permission.^[^ ^49^ ^]^ Copyright 2023, Wiley‐VCH.

Electroacupuncture (EA), which applies controllable electric current to acupuncture needles, has been applied in pain management,^[^ ^34^, ^35^, ^36^ ^]^ neurological diseases,^[^ ^37^, ^38^, ^39^ ^]^ tissue regeneration,^[^ ^40^, ^41^ ^]^ obesity control^[^ ^42^ ^]^ and inflammation regulation.^[^ ^43^, ^44^, ^45^, ^46^ ^]^ Studies have demonstrated that EA could activate different neural pathways in a voltage, frequency, and depth‐dependent manner to achieve systemic anti‐inflammatory effects.^[^ ^6^, ^9^, ^44^ ^]^ For example, Liu et al. showed that high‐intensity electrical stimulation at acupoints ST25 and ST36 induced a spinal sympathetic reflex independent of ProKR2Adv neurons. In contrast, low‐intensity electrical stimulation required deeper stimulation at the ST36 site to drive the vagal‐adrenal axis (**Figure** **2**a–e).^[^ ^13^ ^]^ With the profound research on the mechanism of EA and the support of neuroanatomy in recent years, its application has been increasingly expanded with optimized electrostimulation parameters. In addition to exerting therapeutic effects through electrical and mechanical stimulation, advanced researches have been explored to leverage EA‐generated electrochemistry and electrocatalysis for the treatment of cancer.^[^ ^47^, ^48^, ^49^ ^]^ Qi et al. reported an imaging‐guided endogenic H_2_‐augmented electrochemo‐sonodynamic co‐therapy, in which Fe needles served as electrodes directly at the tumor site (Figure 2f–i).^[^ ^49^ ^]^ Given the acidic tumor microenvironments (TME), H_2_ could be controllably produced in situ, exerting cytotoxic effects on tumor cells through the hydrogen hydrants effect. Additionally, the generated gas bubbles functioned as contrast agents to enhance tumor imaging while simultaneously facilitating localized hyperthermia (44 °C) under ultrasound stimulation to further disrupt tumor tissue. This dual‐mode imaging and therapeutic platform provides a feasible strategy for achieving precise treatment of tumors. In another work, Liu et al. proposed an acupuncture‐based electrocatalytic treatment by modifying a NiO‐P_700_ electrocatalyst layer with optimized atomic spacing (4.1762 Å) on the surface of silver acupuncture needles.^[^ ^48^ ^]^ This configuration enabled an exogenous generation of 0.6 µmol L^−1^ reactive oxygen species (ROS) at the tumor site, yielding promising results in treating hepatocellular carcinoma. Similarly, leveraging the ability to produce ROS in situ, Martin Fussenegger's team utilized EA as a switch to drive electrogenetic regulation of target genes, demonstrating its functionality in a type 1 diabetic mouse model.^[^ ^50^ ^]^ By subcutaneously implanting engineered cells that respond to ROS generated by low‐voltage direct current and modulate insulin expression, EA enabled metabolic intervention in a time‐/voltage‐dependent manner. A daily 10‐second direct current stimulation (4.5 V) achieved stable blood glucose levels for 24 h. This technology holds potential for integrated detection, intervention, and real‐time drug delivery, offering promising prospects for the development of wearable electronic devices capable of directly modulating gene expression.

Given the high cost and potential electrical hazards associated with commercial EA power supply devices, researchers have actively sought to enhance EA equipment for safer and more cost‐effective applications. In this regard, Wei et al. designed a freestanding rotary triboelectric nanogenerator (FR‐TENG) based EA device, which not only maintained a sufficient current output but also featured a compact design and reduced production costs.^[^ ^38^ ^]^ With the assistance of TENG technology, bidirectional continuous current was applied to acupoints (GV14 and GV4) of rats through milli‐needles. Compared with traditional unidirectional current, this bidirectional current could maintain charge balance and mitigate potential side effects for safe and effective EA‐based neurological rehabilitation. In order to further streamline the device and achieve self‐powering, Hu and co‐workers integrated TENG sensor onto the coiled head of an acupuncture needle.^[^ ^51^ ^]^ This sensor comprised a triboelectric layer, an insulating layer, and a copper conductive layer. By incorporating multi‐walled carbon nanotubes into the polydimethylsiloxane triboelectric film, the charge‐loading capacity was significantly enhanced, enabling the generation of a stable open‐circuit voltage output (±6 V) and a short‐circuit current (±225 pA) with an applied force of 10 N. This design allowed acupuncture needles to convert mechanical energy to electrical stimulation through nature treatment motions, such as pinching, lift and thrust motion, as well as rotation around the acupuncture axis.

## 3 Therapeutic‐Enhanced EANs

As a supplementary and alternative therapeutic approach, acupuncture plays a pivotal role in mitigate the adverse effects of pharmacological treatments, especially chemotherapy drugs.^[^ ^52^, ^53^, ^54^ ^]^ Furthermore, acupuncture could optimize therapeutic outcomes by improving drug penetration and absorption while modulating the local microenvironment, making it a promising strategy for integrative medicine.^[^ ^55^ ^]^

### 3.1 EAN‐Promoted Tissue Regeneration

**Figure 3:**
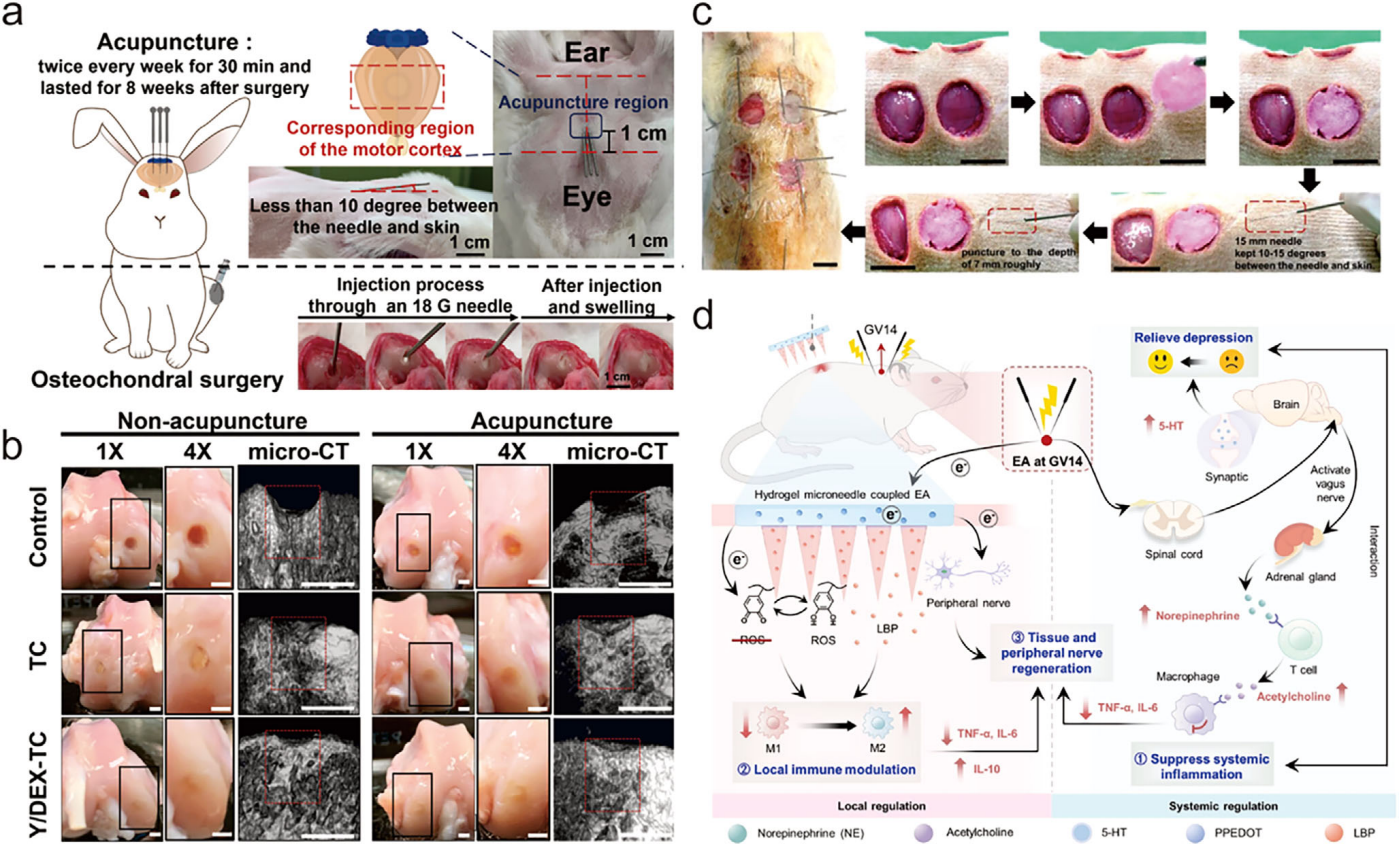
Examples of therapeutic enhanced EANs. a) Schematic diagram of the procedure of animal experiment and location for the acupuncture points. b) The macroscopic appearance and micro‐CT images of the regenerated tissues in different groups after 8 weeks post operation, sacle bar: 1 cm. Adapted with permission.^[^ ^57^ ^]^ Copyright 2024, Wiley‐VCH. c) The procedure and location for the acupuncture points surrounding the wounds. Adapted with permission.^[^ ^58^ ^]^ Copyright 2020, Elsevier Ltd. d) Schematic diagram of synergistic mechanism of hydrogel microneedles integrated with EA at GV14 to treat and enhance repair of skin wounds in diabetic rats. LBP, a natural extract from wolfberry. Adapted with permission.^[^ ^61^ ^]^ Copyright 2024, Elsevier Ltd.

Previous studies have demonstrated that acupuncture can alleviate pain, promote tissue regeneration, and modulate local immune responses.^[^ ^55^, ^56^ ^]^ Leveraging these therapeutic effects, acupuncture has been reported to facilitate wound repair with advanced drug delivery systems. For instance, Chen et al. employed scalp acupuncture for the treatment of osteochondral defects with a drug‐loaded tri‐layer cryogel (**Figure** **3**a,b).^[^ ^57^ ^]^ The acupuncture region were located at the middle area between two eyes and up toward ears of 1 cm, which were considered as the corresponding region of the motor cortex. The acupuncture stimulation enhanced bioelectrical signals, mobilizing endogenous mesenchymal stem cells (MSCs) and promoting their migration to the knee joint injury site, where a cryogel was injected. The cryogel made of chitosan and difunctionalized polyurethane was loaded with Rho‐kinase inhibitor Y27632 and dexamethasone, serving as a bioactive scaffold to support the proliferation and differentiation of recruited MSCs. In an osteochondral defects model of rabbits, this cryogel‐featured EAN locally released Y27632 and dexamethasone after insertion to the osteochondral defects, facilitating the migration and mobilization of MSCs into the cryogel scaffold, where they underwent chondrogenesis or osteogenesis for accelerated osteochondral regeneration. Additionally, acupuncture treatment promoted the M2/M1 macrophage ratio (1.5‐fold), and exhibited improved osteochondral regeneration as evidenced by the higher bone volume fraction (60%) compared to the non‐acupunctured group (52%).

In another work, Chen et al. investigated the synergistic effects of acupuncture‐assisted therapy and adipose‐derived stem cell‐seeded cryogel/hydrogel biomaterials in promoting wound healing in a diabetic rat model (Figure 3c).^[^ ^58^ ^]^ Acupuncture was applied around the wound area, while the cryogel/hydrogel biomaterials were placed directly over the wound. Cryogel and hydrogel, synthesized from glycol chitosan and a crosslinker bifunctional polyurethane, exhibited complementary properties: cryogel's high porosity facilitated exudate absorption and stem cell recruitment in the early inflammatory phase of wound healing, while hydrogel was used in later stages to support cellular retention and immune response. While the hydrogel can support dermal regeneration, acupuncture can promote blood circulation and re‐epithelialization, which was essential for successful wound healing. Additionally, acupuncture stimulated nitric oxide production, up‐regulated complement levels (C3a and C5a), and reduced M1 macrophages and pro‐inflammatory cytokines (TNF‐*α*, IL‐1*β*). The increased nitric oxide production and complement activation enhanced blood circulation and vessel permeability, facilitating nutrient exchange and inflammatory cell migration. Meanwhile, the modulation of the inflammatory response created a favorable microenvironment for angiogenesis, contributing to accelerated wound healing. Results demonstrated the wounds treated with adipose‐derived stem cell‐seeded biomaterials and acupuncture exhibited faster wound healing rate, with a wound closure rate of 90.6 ± 3.3%, higher than that of adipose‐derived stem cell‐seeded biomaterials without acupuncture (71.8 ± 2.9%). Moreover, acupuncture enhanced granulation tissue formation, as evidenced by a shorter unrecovered epidermis distance (1.4 mm) compared to the non‐acupunctured counterparts (1.53 mm).

Beyond impaired wound healing, diabetes is often accompanied by depression and chronic inflammation, which form a depression‐inflammation pathway that further impedes tissue repair.^[^ ^59^, ^60^ ^]^ In consideration of this issue, Xie and colleagues proposed a microneedle‐EA integrated system, which promoted the healing of chronic wounds while alleviating depression‐like behaviors in diabetic rats (Figure 3d).^[^ ^61^ ^]^ Electrical stimulation at acupoint GV14 suppressed systemic inflammation through the vagal‐adrenal axis and elevated serotonin (5‐HT) and FOXP3 levels. Simultaneously, the hydrogel microneedles, composed of gelatin‐methacryloyl, polydopamine‐modified poly(3,4‐ethylenedioxythiophene) nanoparticles, dopamine, and Lycium barbarum polysaccharide, was applied at the wound site. By leveraging the release of antioxidant components and coupling with electrical signals, this EAN system only modulated the inflammatory microenvironment but also promoted cell proliferation and tissue regeneration. Three days after treatment, the levels of IL‐6 and TNF‐*α* at the wound site significantly decreased than those in the control group (LPS treatment), meanwhile, the granulation tissue gap length in the treatment group (1 mm) was smaller than that in other groups after 21days. This strategy enhanced overall recovery through establishing positive feedback between brain and the local wound site, providing an alternative clinical perspective for diabetic wound management.

### 3.2 EAN‐Facilitated Tumor Treatment

Benefiting from its ability to modulate the TME, acupuncture has been incorporated with pharmacological interventions to enhance cancer treatment.^[^ ^62^, ^63^, ^64^, ^65^ ^]^ For instance, Wan et al. found that peri‐tumoral EA could promote the normalization of blood vessels by inhibiting the expression of Glyoxalase 1, with the optimal effect observed on the third day post‐treatment. This timely integration of EA and chemotherapy improved the overall therapeutic outcome compared with acupuncture or chemotherapy alone, offering a comprehensive medical approach for treating triple‐negative breast cancer. Similarly, immune checkpoint inhibitors have shown limited efficacy against microsatellite‐stable colorectal cancer for most patients due to insufficient immune cell infiltration.^[^ ^66^, ^67^ ^]^ In another research proposed by Wang et al., they highlighted the role of EA in the immunotherapy of microsatellite‐stable colorectal cancer.^[^ ^62^ ^]^ After exploring the optimal current (1.0 mA), authors found that EA could effectively remold TME and sensitize tumors to anti‐programmed death‐1 relying on the stimulator of interferon genes pathway, thereby providing an adjuvant strategy for overcoming resistance in immunotherapy of treating “immune‐desert” tumors.

## 4 Drug‐Delivering EANs

“Acupoint injection” is one of the commonly used techniques in clinical practice, involving the administration of small doses of traditional Chinese and Western medicines into acupoints for disease treatment.^[^ ^68^ ^]^ This procedure generates a synergistic therapeutic effect by stimulating the acupoint during the absorption of the drug, thereby integrating the pharmacological action of the delivered drug with the neuroimmune responses elicited by the physical stimulation of the acupoint.^[^ ^23^, ^69^ ^]^ In this process, the choice of delivered drug is tailored to the specific disease being treated and may include not only small‐molecule anti‐inflammatory agents but also protein‐ or peptide‐based therapeutics. However, traditional acupoint injection encounter several limitations, such as procedural complexity, difficulty in dose control, and pain associated with injections. Based on this, researchers have developed drug‐delivering EANs that integrates mechanical stimulation with direct and local drug delivery in a spatiotemporally synchronized manner. Considering the rigid metal structure of conventional acupuncture needles, drug loading presents a challenge. In order to enhance the drug‐loading capacity of acupuncture, promote drug retention at injury sites, or achieve controlled drug release, numerous studies have engineered acupuncture needles by macroscopic modifications or surface functionalization, thereby improving therapeutic efficacy of EANs.

### 4.1 Macroscopically Customized EANs for Drug Delivering

One feasible approach to modify the acupuncture needles involves macroscopical modifications, such as structural customization or surface etching to facilitate drug loading.^[^ ^70^, ^71^, ^72^ ^]^ For instance, Liu et al. developed a hollow honeycomb‐structured EAN to load melittin for the treatment of rheumatoid arthritis (RA).^[^ ^73^ ^]^ Melittin, a potent anti‐inflammatory agent, was first encapsulated within a pH‐responsive hydrogel composed of chitosan, sodium beta‐glycerophosphate, and hyaluronic acid. The needle was designed with honeycomb structure to enhance drug delivery by integrating a hollow core with porous walls. The hollow core acted as a reservoir for the hydrogel, allowing for high drug‐loading capacity (40 µL), while the porous walls enabled gradual dispersion of the hydrogel, resulting in sustained drug release over 96 h, and the honeycomb structure protected the hydrogel from premature leakage. In RA mice models, the acupuncture needles were inserted at the ST36 acupoint, located ≈3 mm from the ankle joint. The mildly acidic microenvironment of RA‐affected joints triggered the pH‐responsive hydrogel to release melittin, allowing for localized anti‐inflammatory effects. Synergistically, acupuncture modulated physiological processes by stimulating acupoints, enhancing local circulation, and regulating immune responses, thereby creating a favorable environment for drug absorption and action. Results revealed that acupuncture with the honeycomb needles reduced cartilage degradation and synovial inflammation more effectively than drug injection group.

**Figure 4:**
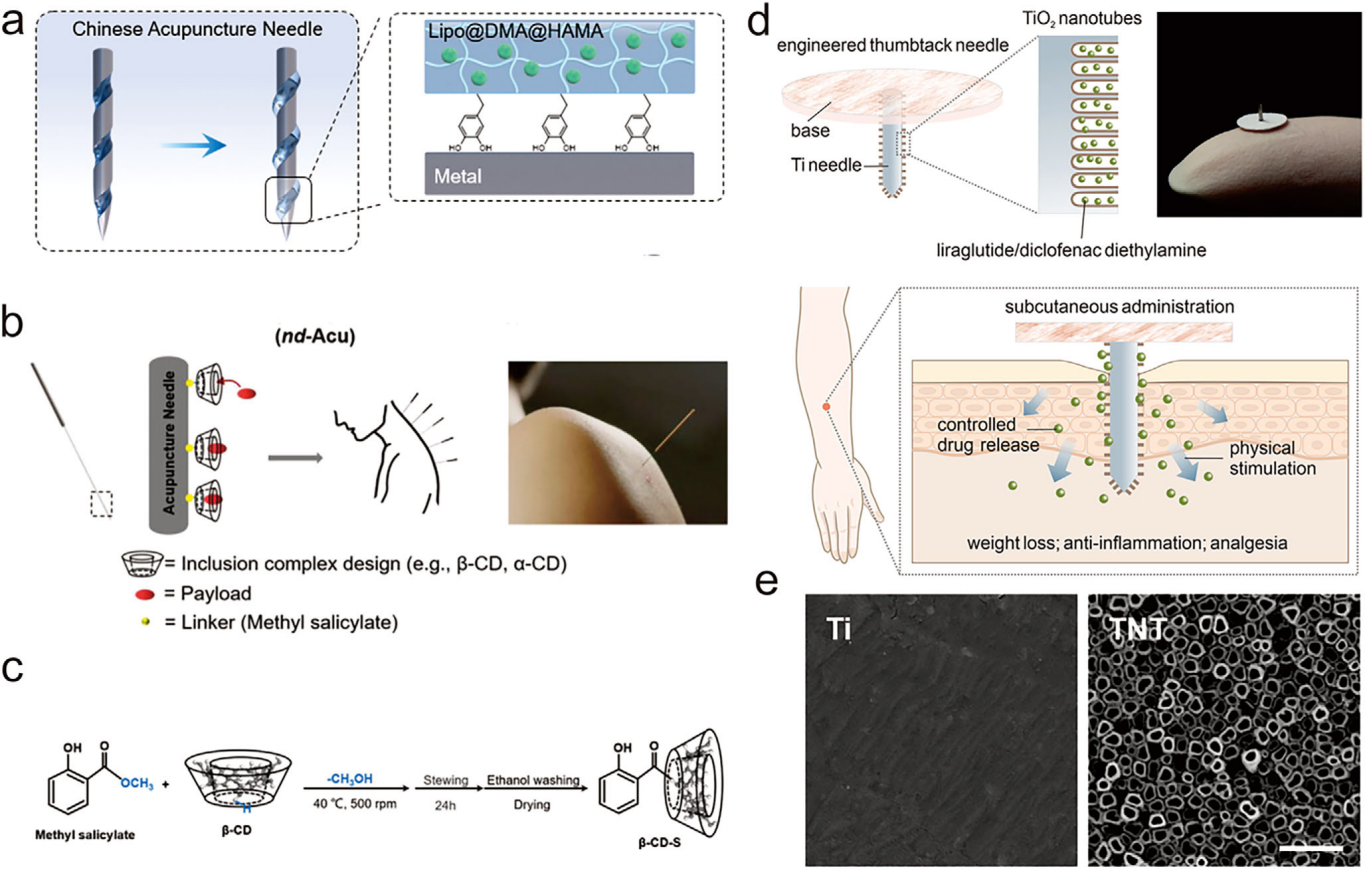
Macroscopic and nano modifications of EANs. a) Chinese acupuncture needles with a screw‐thread structure at the tip designed to penetrate the subchondral bone and transport the hydrogel. DMA, N‐[2‐(3,4‐Dihydroxyphenyl)ethyl]2‐methylprop‐2‐enamide; HAMA, hyaluronic acid methacrylate. Adapted with permission. Adapted with permission.^[^ ^74^ ^]^ Copyright 2022, Wiley‐VCH. b) Schematic of the design of nd‐Acu, showcasing the covalent attachment of a *β*‐CD derivative to the stainless‐steel needle surface. nd‐Acu, nanoenabled drug delivery acupuncture technology; CD, cyclodextrin. c) Chemical reactions for the synthesis of *β*‐CD‐S, the intermediary product for surface deposition on the acupuncture needle. *β*‐CD‐S, *β*‐cyclodextrin salicylate. Adapted with permission.^[^ ^79^ ^]^ Copyright 2023, Wiley‐VCH. d) Scheme illustrating the modified surface‐engineered thumbtack needle for loading of liraglutide and diclofenac diethylamine. e) SEM images of the surface of modified thumbtack needles. Scale bar: 1 µm. TNT, TiO_2_ nanotubes. Adapted with permission.^[^ ^81^ ^]^ Copyright 2024, Elsevier Ltd.

In another study, Lin et al. constructed an EAN with a screw‐thread structure tip for OA treatment. The EAN facilitated effective penetration across the cartilage, and threaded grooves on the needle enhanced the adhesion and retention of the drug‐loaded hydrogel into the subchondral bone (**Figure** **4**a).^[^ ^74^ ^]^ As the EAN was rotated and withdrawn, the hydrogel remained at the target site, ensuring sustained drug (Baicalein) release. In vitro assessments revealed that demonstrated better drug retention in the subchondral bone by the EAN, with 73.25% of the hydrogel delivered, compared to only 29.92% with the conventional acupuncture‐needle. In vivo, drug release persisted for over a month, with ≈50% still present at three weeks. Experiments on OA rats showed that the EAN reduced subchondral bone remodeling and cartilage degeneration, thereby alleviating the symptoms of osteoarthritis. Specifically, the Osteoarthritis Research Society International (OARSI) score decreased by 44.74% in the EAN group, compared to only 19.32% in the Baicalein injection group. Additionally, glycosaminoglycan content in the EAN group reached 66.90%, higher than the 37.77% of Baicalein injection, indicating enhanced cartilage matrix protection. Chondrocyte apoptosis was also reduced, with a 61.31% decrease in the ST‐needle system group versus 40.65% in the Baicalein injection group.

The above system can also be integrated with thermal and electrical stimulation for synergistic therapeutic effects. Lin et al constructed the EAN with thermal‐sensitive hydrogel microspheres for OA treatment.^[^ ^23^ ^]^ The needle (400 µm in diameter, 11 cm in length) featured a screw‐thread groove structure on the tip and a copper wire wrapped around the tail. The thermal‐sensitive hydrogel was composed of hyaluronic acid methacryloyl (HAMA) polymer and drug loaded‐thermosensitive poly(lactic‐co‐glycolic acid)‐poly(ethylene glycol)‐poly(lactic‐co‐glycolic acid) (PLGA‐PEG‐PLGA) nanoparticles. The EAN could successfully break through the cartilage matrix barrier, and the hydrogel then remained in the joint for a month, inhibiting mitochondrial apoptosis and inducing autophagy. Upon heat application, thermosensitive PLGA‐PEG‐PLGA nanoparticles was liquefied, triggering drug release into the knee cartilage. The induced hyperthermia increased deep tissue temperature by 4.5 °C and stimulated HSP‐70 synthesis, reducing chondrocyte apoptosis by 37.23% in a OA rat model. Moreover, the apoptosis rate in the drug delivered‐heat conduction group was significantly lower than that in the heat conduction needle group (61.76%). These findings indicated that the heat enhanced EANs effectively suppresses chondrocyte apoptosis, thereby improving cellular physiological function and alleviating OA progression. Lin et al. embedded gene‐modulating hydrogel microspheres within threaded grooves of the EAN, targeting the organelle network for the treatment of OA.^[^ ^75^ ^]^ This work enabled precise targeting and dual regulation of the organelle network, exhibiting fewer apoptotic chondrocytes in vivo compared with the group treated with acupuncture alone.

Beyond efficacy enhancement, drug‐delivering ENA can also mitigate potential side effects. For instance, “electrical overload” remains a challenge in EA therapy, arising from heterogeneous current distribution. This imbalance can cause cellular damage and impede tissue regeneration in the core region. To alleviate the adverse effects of electrical overload, Cui and coworkers integrated localized drug delivery system with EA for the treatment of peripheral nerve injury.^[^ ^76^ ^]^ By surface coating of polydopamine and chitosan onto the screw‐structured needle, the EAN enabled effective loading of vitamin‐encapsulating lipid microspheres. Upon insertion along the damaged sciatic nerve in a rat model, the administered EA therapy enhanced the total antioxidant capacity of the tissue by 78.5%, demonstrating optimal pro‐synaptic regeneration. While the locally delivered microspheres by the EAN help reduced ROS by 72.8% and apoptosis by 59.5% under the electrical stimulation. Compared with local drug injections aimed at counteracting electrical overload, this approach enabled more precise and synchronized mitigation of oxidative stress‐induced damage, enhancing the safety and efficacy of EA therapy.

### 4.2 Nano/Micro‐Modified EANs for Drug Delivering

In addition to macroscopical customization, surface engineering technologies offer another strategy of modifying medical devices for drug adsorption while preserving their structural integrity and mechanical properties.^[^ ^68^, ^77^ ^]^ For instance, Wang et al. developed a diclofenac sodium (DS) loaded EAN through a multilayer modification process for the RA treatment.^[^ ^78^ ^]^ DS was encapsulated within the needle's surface layers, which were constructed through a layer‐by‐layer self‐assembly technique driven by electrostatic forces. The resulted membrane, consisted of alternating layers of poly(styrenesulfonate) and poly(allylamine hydrochloride), was ≈0.55 µm thick. These layers functioned as a stable reservoir, enabling sustained DS release from the EAN at acupoints (ST36 and BL60). In vivo studies on RA rat models revealed that the system exhibited a 9.4‐fold increase in plasma concentration compared to oral administration. Compared with intra‐articular injection or intragastric administration, the DS‐loaded EAN exhibited more effective alleviation of pain and joint swelling in RA rats.

Researchers also developed a nano‐enabled drug delivery acupuncture technology (*nd*‐Acu) with nano‐sized cavity modified on the surface of needles for OA treatment (Figure 4b,c).^[^ ^79^ ^]^ In detail, this approach utilized an electrochemical procedure to functionalize the stainless‐steel needle surface with methyl salicylate‐modified cyclodextrin. These cyclodextrins are molecules with a nano‐sized, hydrophobic cavity that can encapsulate drugs, such as lidocaine, forming an inclusion complex. This nano‐scale encapsulation process allows for precise drug loading and release at the acupuncture site. In a knee OA model of mice, lidocaine‐loaded *nd*‐Acu was applied at ST36 and GB34 acupoints for 30 min. Results showed that the treatment alleviated inflammation, as evidenced by a significant reduction in pro‐inflammatory cytokines IL‐1*β* and IL‐6 in the articular cavity compared to lidocaine acupoint injection and triamcinolone acetonide intra‐articular injection. Additionally, lidocaine‐loaded *nd*‐Acu exhibited better protective effects on joint integrity, mitigating cartilage proteoglycan depletion and reducing the OARSI score compared to acupuncture alone or lidocaine injection.

Although effective, traditional acupuncture needles (typically 1.5–13.5 cm) may cause discomfort and require precise insertion by licensed acupuncturists. To improve compliance and simplify application, researchers have developed miniaturized alternatives. Thumbtack needles are a common type of intradermal acupuncture, which use relatively short needles (0.3–2.5 mm) for sustained stimulation at superficial acupoints. This less invasive approach enhances patient comfort while maintaining continuous stimulation for therapeutic efficacy.^[^ ^80^ ^]^ However, thumbtack needle remain limited by suboptimal therapeutic efficacy and prolonged treatment duration. For instance, Cai et al. developed a surface‐engineered thumbtack needle for minimal invasiveness while providing sustained stimulation to acupoints (Figure 4e,d).^[^ ^81^ ^]^ These EANs, made from titanium (Ti), underwent anodization to generated porous titanium oxide nanotubes on the surface. These nanotubes, with diameters ≈100 nm and lengths of ≈1.1 µm, increased the surface area for improved drug loading‐capacity. Depending on therapeutic requirements, the drug release profiles of thumbtack needles can be customized by self‐assembled monolayers or coatings onto the nanotube‐modified EAN in a type 2 diabetes mellitus mouse model, liraglutide (LRT)‐loaded thumbtack needles effectively regulated plasma glucose levels (<260 mg dL^−1^), reduced weight (<43 g compared with 53 g in the control group), and significantly improved insulin sensitivity to ≈48.5 (≈117.6 in LRT injection group) over a five‐week treatment period. Similarly, in an OA rat model, diclofenac diethylamine‐loaded needles were administrated at acupoints of ST35, ST36, and GB34, which alleviated pain hypersensitivity and reduced joint inflammation. Compared to the acupuncture group or the drug administration group, both drug‐delivery EANs demonstrated enhanced therapeutic effects and reduced drug resistance or other side effects.

## 5 Challenges and Future Perspectives

The development of EANs integrates advanced materials science, macro‐/nano‐engineering, and biomedical instrumentation with traditional Chinese medicine. By incorporating electrical stimulators, heating components, and drug‐delivering capabilities, EANs enhance therapeutic efficacy while minimizing potential side effects in cancer therapy, wound healing, diabetes management, and osteoarthritis treatment. These advancements transform traditional acupuncture needles into intelligent therapeutic tools with enhanced precision and effectiveness. **Table** **1** summarizes recent innovations in EANs, detailing advancements in needle materials, fabrication techniques, integrated functionalities, targeted acupoints, and **Table** **2** summarizes their corresponding clinical applications.

**Table 1:** Summary of representative EANs and their applications. NA: not applicable.

Type of EANs	Material of EANs	Engineering techniques	Function of EANs	Disease	Administrative regions	Ref
WA	silver; stainless steel	acupuncture needle; affixed with moxa or other heating supply system	inducing heat stimulation; reducing inflammatory factors; alleviating pain	OA	ST25, ST36, et al.	[27]
EA	stainless steel	acupuncture needle (0.16 × 7 mm); equipped with power supply system	inducing electrical stimulation; reducing inflammatory factors	systemic inflammatory response syndrome	ST25, ST36	[13]
	Iorn (Fe)	acupuncture needle (0.35 × 40 mm); equipped with power supply system	inducing hydrogen therapy; promoting tumor cell apoptosis	breast cancer	tumor site	[49]
	silver	acupuncture needle with a NiO‐P700 electrocatalyst layer; equipped with power supply system	generating ROS; promoting tumor cell apoptosis	hepatocellular carcinoma (HCC)	tumor site	[48]
	stainless steel; Platinum (Pt)	Pt and poly(3,4‐ethylenedioxythiophene) polystyrene sulfonate (PEDOT:PSS) deposited acupuncture needles; equipped with power supply system with steady direct current	regulating engineered human cells (hMSC‐TERT cell clone DC_INS_) to release insulin	type1 diabetes	dorsum of mice	[50]
	stainless steel	FR‐TENG based acupuncture needle (0.3 × 30 mm);	enhancing neuron survival in the ventral horn; inhibiting astrocyte activation at the lesion site; promoting locomotion functional recovery	spinal cord injury	GV14, GV4	[38]
	silver	acupuncture needle (0.25 × 25 mm) integrated with TENG sensor	optimizing EA application	NA	NA	[51]
therapeutically enhanced EAN	stainless steel	acupuncture needle (0.25 × 15 mm)	promoting tissue regeneration	osteochondral defects	the middle area between two eyes and up toward ears of 1 cm	[57]
	stainless steel	acupuncture needle (0.25 × 15 mm)	facilitating wound healing	the diabetic skin wound	around the wound area	[58]
	stainless steel	acupuncture needle	inducing electrical stimulation; reducing inflammatory factors; regulating monoamine neurotransmitter (5‐HT) expression and enhancing the restoration of synaptic plasticity	diabetes‐related compliances and emotional disorders	GV14	[61]
	stainless steel	acupuncture needle (0.18 × 15 mm)	promoting the normalization of blood vessels	triple‐negative breast cancer	tumor site (≈5 mm off the boundary in the up, down, left, and right directions of the tumor)	[65]
drug delivering EAN	314 medical‐grade stainless steel	hollow honeycomb acupuncture needles (0.6 × 50 mm) featured with four rows of pores and carried with melittin‐loaded hydrogel	drug delivery; reducing inflammatory factors; modulating RA immune function	RA	ST36	[73]
	stainless steel	acupuncture needle with a screw‐thread structure tip (tip diameter ≈ 200 µm, length 10–15 cm); carried with baicalein‐loading Lipo embedded hydrogel	penetrating the physical barrier; targeted drug delivery; enhancing the adhesion and retention of the drug‐loaded hydrogel; reducing inflammatory factors	OA	knee joint	[74]
	stainless steel	acupuncture needle (400 µm × 11 cm) with a screw‐thread groove structure on the tip; carried with thermal‐sensitive hydrogel loaded with nanoparticles containing PARKIN protein; equipped with heating supply system	inducing heat stimulation; penetrating the physical barrier; targeted drug delivery; reducing inflammatory factors	OA	knee joint	[23]
	stainless steel	screw‐threaded acupuncture needle (400 µm × 11 cm); carried with thermal‐sensitive hydrogel loaded with nanoparticles containing small interfering RNA; equipped with power and heating supply system	inducing electrical stimulation; penetrating the physical barrier; targeted drug delivery; reducing inflammatory factors; improving the activities of the lesion cells	OA	knee joint	[75]
	stainless steel	screw‐structed acupuncture needle (2 mm in length); carried with microsphere hydrogel loading VB12 and VE succinate	targeted drug delivery; improving uneven electrical stimulation effects; promoting neuroregeneration	peripheral nerve injury	core region experienced “electrical overload”	[76]
	stainless steel	acupuncture needle (0.30 × 40 mm) modified with diclofenac sodium (DS)‐loaded multilayers	drug delivery; reducing inflammatory factors; alleviating pain and joint swelling	RA	ST36, BL60	[78]
	stainless steel	acupuncture needle modified with nano‐sized cavity on surface by *nd*‐Acu technology and loaded with lidocaine	drug delivery; reducing inflammatory factors; slowing down KOA development biochemically	KOA	ST36, GB34	[79]
	Ti; TiO_2_	thumbtack needle (0.3 × 2.5 mm) modified with TiO2 nanotubes on surface; with self‐assembled monolayers of (3‐aminopropyl)‐triethoxysilane for loading of liraglutide; or with coating of diclofenac diethylamine‐loade polyvinyl alcohol layers	controlled drug delivery; sustained transdermal stimulation; reducing inflammatory factors; regulating plasma glucose level; reducing drug resistance	OA; type 2 diabetes	GB 34, ST 36, ST 35; BL 20, BL 23, ST 36, SP 6	[81]

**Table 2:** Summary of representative clinical studies involving·EAN·systems from clinicaltrial.gov. NA: not applicable.

Type of EANs	Recruitment Status	Phase	Start date/ completion date	Study target	Design	Subjects	Trial identifier
EA	Completed	1,2	2007/2010	To test whether EA as an adjunctive treatment can improve outcomes among patients receiving inpatient opioid detoxification from opioids.	Allocation: randomized; Masking: single (participant); Participant group/arm: active EA/ sham EA; Acupoints: LI4/ P8 on one hand and P6/ TE 5 on the opposite arm.	Age: 18 years to 59 years patients receiving inpatient detoxification from opioids; Sex: all sexes; N = 48.	NCT00742170
	Completed	2	2004/2007	To compare the safety, efficacy, and tolerability of EA and sham EA for the treatment of major depression.	Allocation: randomized; Masking: single; Participant group/arm: EA/ sham EA; Acupoints: GV‐20‐on the crown of the head and yin tang‐on the midline of the forehead.	Age:18 years to 80 years adults with a major depressive disorder of mild or moderate severity; Sex: all sexes; N = 57.	NCT00071110
	Completed	NA	2013/2016	To evaluate the efficacy and safety of EA for symptoms of women during menopausal transition.	Allocation: randomized; Masking: double (participant, outcomes assessor); Participant group/arm: EA/ sham EA; Acupoints: RN4, EX‐CA1, ST25, SP6 (double sides).	Age: 40 years to 55 years adults; Sex: female; N = 360.	NCT01849172
	Completed	NA	2017/2017	To explore the effectiveness and safety of EA therapy for the stroke survivors with shoulder pain.	Allocation: randomized; Masking: double (participant, outcomes assessor); Participant group/arm: verum EA/ sham EA; Acupoints: LI4, LI15, TE14, SI9, SI11, and GB21, unilaterally.	Age: 19 years and older stroke survivors; Sex: all sexes; N = 45.	NCT03086863
	Completed	NA	2015/2017	To assess the efficacy of EA on pain control, perception of pain, plasma cortisol and beta‐endorphins levels, patient‐perceived quality of life and use of pain medications, in people with chronic knee pain.	Allocation: randomized; Masking: triple (participant, investigator, outcomes assessor); Participant group/arm: EA/ sham acupuncture; Acupoints: local points St 34, St 35, St 36, Liv 8, Sp 10 and one distal point St 44.	Age: 50 years to 80 years adults; Sex: all sexes; N = 160.	NCT02299713
	Recruiting	NA	2025/2026	To evaluate the efficacy and safety of EA combined with standard Intensive Care Unit therapy on organ dysfunction and other clinical outcomes in sepsis patients.	Allocation: randomized; Masking: double (participant, outcomes assessor); Participant group/arm: EA/ sham EA; Acupoints: ST36 and GB34.	Age: 18 years and order sepsis patients; Sex: all sexes; N = 308.	NCT06666946
WA	Completed	2	2015/2018	To evaluate the effectiveness and safety of warm acupuncture for allergic rhinitis.	Allocation: randomized; Masking: single (outcomes assessor); Participant group/arm: WA/loratadine taken orally; Acupoints: LI 4, LI 20, LU 5, ST 2, EX‐HN 3, EX‐HN 8, GV 23, Du 14.	Age: 18 years to 60 years adults Sex: all sexes; N = 98.	NCT02339714
	Completed	NA	2022/2023	To observe the safety and effectiveness of warm acupuncture in the treatment of cold‐sensitive fibromyalgia patients.	Allocation: randomized; Masking: single (outcomes assessor); Participant group/arm: WA/ without any intervention; Acupoints: NA.	Age: 18 years and order adults Sex: female; N = 38.	NCT05228990
	Completed	NA	2018/2022	To indicate the effects of acupuncture, electro‐acupuncture, moxibustion, warm‐needling, sham‐needle, Celebrex treatments for knee osteoarthritis.	Allocation: randomized; Masking: single (participant); Participant group/arm: acupuncture/ EA/ moxibustion/ WA/ sham‐needle/ Celebrex; Acupoints:ST 34, SP 10, EX‐LE 4, ST 35, GB 34, SP 9.WA are applied at EX‐LE 4 and ST 35.	Age: 40 years to 75 years adults Sex: all sexes; N = 360.	NCT03563690
Acupuncture combined with drugs	Completed	2	2006/2007	To evaluate the efficacy of rosiglitazone and EA combined therapy on patients with type 2 diabetes mellitus.	Allocation: randomized; Masking: single (participant); Participant group/arm: EA + Rosiglitazone/ Rosiglitazone; Acupoints: NA.	Age: 20 years to 65 years patients with type 2 diabetes mellitus; Sex: all sexes; N = 49.	NCT01577095
	Recruiting	3	2024/2027	To observe the safety and efficacy of Specific Mode EA Stimulation (SMES) combined with albumin‐bound paclitaxel (ABX) in treating patients with recurrent high‐grade gliomas postoperatively.	Allocation: randomized; Masking: triple (participant, care provider, outcomes assessor); Participant group/arm: Temozolomide (TMZ)/ SMES+ABX+TMZ; Acupoints: GV20 and GV26.	Age: 18 years to 70 years postoperative glioma patients; Sex: all sexes; N = 58.	NCT06330337
	Completed	1	2013/2016	To study the feasibility of a novel treatment for women with provoked localized vulvodynia with acupuncture and 5% lidocaine cream.	Allocation: randomized; Masking: double (participant outcomes assessor); Participant group/arm: acupuncture + lidocaine/ non‐ acupuncture + lidocaine; Acupoints: NA.	Age: 18 years to 45 years adults. Sex: female; N = 19.	NCT01996384
	Completed	NA	2015/2017	To evaluate the comparative clinical effectiveness of pharmacopuncture for severe non‐acute sciatic pain patients diagnosed with lumbar disc herniation with usual care of conventional medicine and acupuncture.	Allocation: randomized; Masking: triple (participant, care provider, outcomes assessor); Participant group/arm: Shinbaro pharmacopuncture/acupuncture/ usual care; Acupoints: EX B2, GB30, BL40, BL25, BL23, GB34.	Age: 25 years to 65 years severe non‐acute sciatic pain patients; Sex: all sexes; N = 60.	NCT02384928

Despite these advancements, several challenges remain in their development and clinical application. First, the design of EANs still faces several limitations and necessitates the integration of further advanced technologies. For instance, miniaturizing and improving the portability of EANs remains a major hurdle, as current devices often rely on bulky external power sources that limit mobility and restrict treatment areas. The integration of microelectronics and flexible electronics could enhance wearability and patient compliance. Moreover, optimizing the drug‐loading capacity of EANs is essential to meet clinical dosing requirements. Due to the limited size of acupuncture needles, engineering strategies such as macro‐scale etching and nano‐surface modifications are necessary to enhance drug retention and controlled release. Drawing on microneedle delivery platform strategies, the use of biocompatible polymers as the primary material and micro‐molding casting techniques can also be applied to the development of novel drug‐loaded acupuncture needles, aiming to achieve high drug loading capacity and physiologically responsive smart drug delivery.

Secondly, for the safety and ethical considerations for drug‐loaded EANs, the controllability of drug release is unstable, with potential risks including drug accumulation at the local site after multiple needle insertions and the residuals of needle materials. These factors necessitate long‐term and rigorous sampling and testing. Ethically, patients need transparent communication of risks, including informing them of the additional risks associated with drug‐loaded acupuncture needles compared to traditional acupuncture, such as potential drug allergies.

Thirdly, for the regulation of the clinical translation, standardizing needle insertion techniques is crucial for reducing variability among practitioners. Real‐time feedback mechanisms and point‐of‐care detection technologies could improve precision and consistency in acupuncture treatment.^[^ ^82^, ^83^ ^]^ Besides, standardization requires further refinement. Clinically, selecting acupoints along the meridians corresponding to the disease site is the primary method of acupoint selection, with a wide variety of combination strategies.^[^ ^84^ ^]^ Although Ma cowokers^[^ ^13^ ^]^ have demonstrated the acupoint selectivity and specificity of ST36 in activating the vagus–adrenal axis, the specificity of individual acupoints, stimulation intensity, needling depth, and outcome measurements still require further investigation and standardization. Moreover, there is a lack of unified standards for the current dosage in electroacupuncture and the thermal dosage in warm acupuncture, which hinders the consistent evaluation of therapeutic efficacy.

So far, although the meridian theory of acupuncture is difficult to fully verify through existing biomedical models, an increasing number of studies have validated and summarized the neuroimmunology principles of acupuncture.^[^ ^11^ ^]^ Based on this, EANs integrating customized drug delivery and augmented physical stimulation hold significant potential in chronic disease management, rehabilitation medicine, and precision healthcare. Nevertheless, EANs hold promising potential in chronic disease management, rehabilitation medicine, and precision healthcare. Their adaptability allows for personalized treatment tailored to specific diseases and targeted lesions. Additionally, the portability of these EANs facilitates home‐based therapy, enhancing improving adherence to long‐term treatment regimens. Furthermore, the integration of biosensors with EANs could enable real‐time monitoring and adaptive treatment adjustments, forming an intelligent, self‐feedbacked acupuncture system. With the deepening intersection of materials science, artificial intelligence, and traditional Chinese medicine, EANs have the potential to evolve as an efficient intelligent medical devices.

## Conflict of Interest

Z.G. is the co‐founder of Zenomics Inc. and ZCapsule Inc. Z.G. and Y.Z. are the co‐founders of *µ*Zen Pharma Co., Ltd., and the other authors declare no conflict of interest.
